# Novel Polyheteroarylene Membranes for Separation of Methanol‒Hexane Mixture by Pervaporation

**DOI:** 10.1038/s41598-018-36118-4

**Published:** 2018-12-14

**Authors:** G. Polotskaya, A. Pulyalina, M. Goikhman, I. Podeshvo, V. Rostovtseva, S. Shugurov, I. Gofman, N. Saprykina, N. Gulii, N. Loretsyan, A. Toikka

**Affiliations:** 1 Department of Chemical Thermodynamics and Kinetics, Saint Petersburg State University, Saint Petersburg, 198504 Russia; 2 Institute of Macromolecular Compounds, Russian Academy of Sciences, Saint Petersburg, 199004 Russia

## Abstract

Polymer membranes with improved transport properties are required for effective separation of organic mixtures (such as methanol‒hexane system) by combination of pervaporation and azeotropic distillation. The present work is devoted to comparative study of two types of membranes based on poly(amidoimide acid) with 2,2′-biquinoline-6,6′ units in the backbone; the objects were prepared (i) from the initial polymer and (ii) from the polymer-metal complex (with Cu(I)). Thermo-mechanical and mass spectrometric investigations demonstrated good operational properties of the samples. Density measurements and SEM analysis revealed that the structure formed in polymer-metal complex is more compact as compared to that of the pure polymer membrane. Mass transfer processes of methanol and hexane through both kinds of membranes were studied by sorption, desorption and pervaporation tests. The values of equilibrium sorption degree, the Flory–Huggins parameter, and diffusion coefficient were determined for the obtained membranes. The pervaporation data allowed calculating permeability and selectivity of membranes in addition to the flux and the separation factor. The membrane based on polymer-Cu(I) complex allowed separating the methanol‒hexane azeotropic mixture with a separation factor of 980 and pervaporation separation index equal to 66.6; therefore, this process is significantly more effective than separation procedures involving other known membranes.

## Introduction

Modern membrane technologies have many environmental and economic advantages over traditional methods of purification, concentration, and separation of different substances^1,2^. Membrane pervaporation is used to separate liquid mixtures and involves partial vaporization through a nonporous membrane^3,4^. The driving force of pervaporation is a difference between chemical potentials; this difference corresponds to the concentration gradient existing between liquid and vapor phases on the opposite sides of a membrane. The permeated component in vapor form is collected on the side of a membrane where low pressure is applied^5^. Pervaporation membranes should possess enhanced permeability and selectivity combined with excellent durability. Polymer membranes are of particular interest for these applications because they offer such advantages as reasonable economic viability, ease of fabrication and large-scale production^6–8^.

Pervaporation is a promising technique for separation of liquid mixtures; this process frequently replaces distillation^9–12^. In certain complicated cases, a combination of the two above mentioned methods, i.e., distillation and pervaporation, is desirable or required. There are various approaches for combining both processes, e.g., see^13^. Pervaporation may be combined with azeotropic distillation^14^. In such hybrid process, the pervaporation stage helps improve purity of the top and bottom products of distillation^15^.

Methanol is one of the common and widely used organic solvents; thus, isolation of methanol from its mixtures is of great practical importance. Due to the fact that a number of solvents have almost similar boiling points to that of methanol, and since methanol forms azeotropes with a lot of liquids like bromomethane, propanone-2, benzene, heptane, etc., it is difficult to separate such mixtures by conventional distillation^16^. Accordingly, the process of azeotropic distillation can be applied for methanol separation and purification with suitable separation agents. The data on methanol containing azeotropic mixtures can be found in the literature; the azeotropic mixture with the lowest boiling point of 49.5 °C (760 mm Hg) consists of 27.1 wt% of methanol and 72.9 wt% of hexane^17^. Therefore, hexane can be an effective entrainer for isolating methanol by azeotropic distillation. Hexane is added to separate the methanol/acetone mixtures that are formed in the production of hydrocarbons and during destructive distillation of wood^18^. Hexane is also used in azeotropic distillation of the methanol/tetrahydrofuran mixture that is formed during production of poly(butylene terephthalate)^19^. The final stage (separation of methanol/hexane azeotropic mixture) can be performed by the modern technique of membrane pervaporation.

In membrane processes, much attention is paid to selection of the appropriate membrane materials for separation of every specific mixture^20^. Unfortunately, the literature on pervaporation of methanol‒hexane mixtures is very scarce. Cabasso *et al*.^21^ studied Nafion ion-exchange hollow fibers that had a separation factor of 17 and a flux of 0.58 kg/m^2^ h in pervaporation of the azeotropic methanol‒hexane mixture. Genduso *et al*.^17^ studied PolyAnTypM1 membrane (the polyacrylonitrile ultrafiltration membrane with pores filled with a functional polymer) whose separation factor did not exceed 19, and the flux was 3.8 kg/m^2^ h in pervaporation of the mixture containing 84 wt% of methanol. Thus, the known membranes intended for separation of binary methanol-hexane mixtures are far from perfect, and new effective membrane materials are required for this purpose.

Among the most demanded membrane materials, of special interest are the polymers of heteroaromatic structure that exhibit high mechanical strength, thermal stability, and chemical resistance^22–27^. In the recent years, polyheteroarylenes containing imide and oxazinone fragments in the backbone have attracted attention of researchers as membrane materials for gas separation and pervaporation. Two main polymers of this group of polyheteroarylenes, namely, imide-containing polyamic acids and polybenzoxazinoneimides, have been studied in native form and as polymer-metal complexes with copper (Cu(I))^24–28^. Application of polymer-metal complexes as membrane materials offers interesting prospects in membrane processes. Use of these complexes allows purposeful changing polymer properties, which depend on composition of fragments of a complex, positions of substituents in the ligand group, and architecture of the coordination link.

In our previous works, polymer-metal complexes based on monomers containing biquinoline and carboxylic groups, namely, 2,2′-biquinoline- 4,4′-, 6,6′-, 7,7′-, and 8,8′-dicarboxylic acids, have been studied^25–28^. Films of these polymer-metal complexes have proven themselves to be suitable membranes for gas separation and pervaporation. In some cases, in order to improve transport properties, polymers with increased chain flexibility are required. However, the polymer chains containing biquinoline‒dicarboxylic acid units have relatively high rigidity, since only an amide group and a single bond between two biquinoline rings can be considered as hinge units that allow changing polymer conformation.

The uniqueness and novelty of the polymer chosen for membrane preparation in the present work lie in the fact that its macromolecular chain contains biquinoline-dimethanamide groups. The new bifunctional biquinoline-containing monomer ([2,2′-biquinoline]-6,6′-diyldimethanamine) was used as a starting compound. The presence of flexible methylene groups in the backbone increases the number of conformations in such macromolecules and thereby reduces polymer chain rigidity.

The object of the study in the present work was a polyheteroarylene containing imide, amide, and carboxylic groups, with 2,2′-biquinoline-6,6′ units in the backbone (the so-called polyamidoimide acid (PAIA)). Comparative study of two membranes based on PAIA in its native form and in the form of a complex between the polymer and copper ions (PAIA-Cu(I)) was carried out for the first time. Accordingly, the aim of our work was to study the pervaporation of methanol‒hexane mixture using the PAIA and PAIA-Cu(I) membranes, and to analyze transport properties of novel membranes on the basis of the obtained information about their structure and physicochemical characteristics.

## Results and Discussion

In this work, samples of PAIA and PAIA-Cu(I) containing amide, methylene, carboxylic, ether, imide, and biquinoline groups in the polymer chain were synthesized. Chemical structure of the polymers was confirmed by ^1^H NMR spectroscopy.

### Physico-mechanical properties and structure of membranes

**Figure 1:**
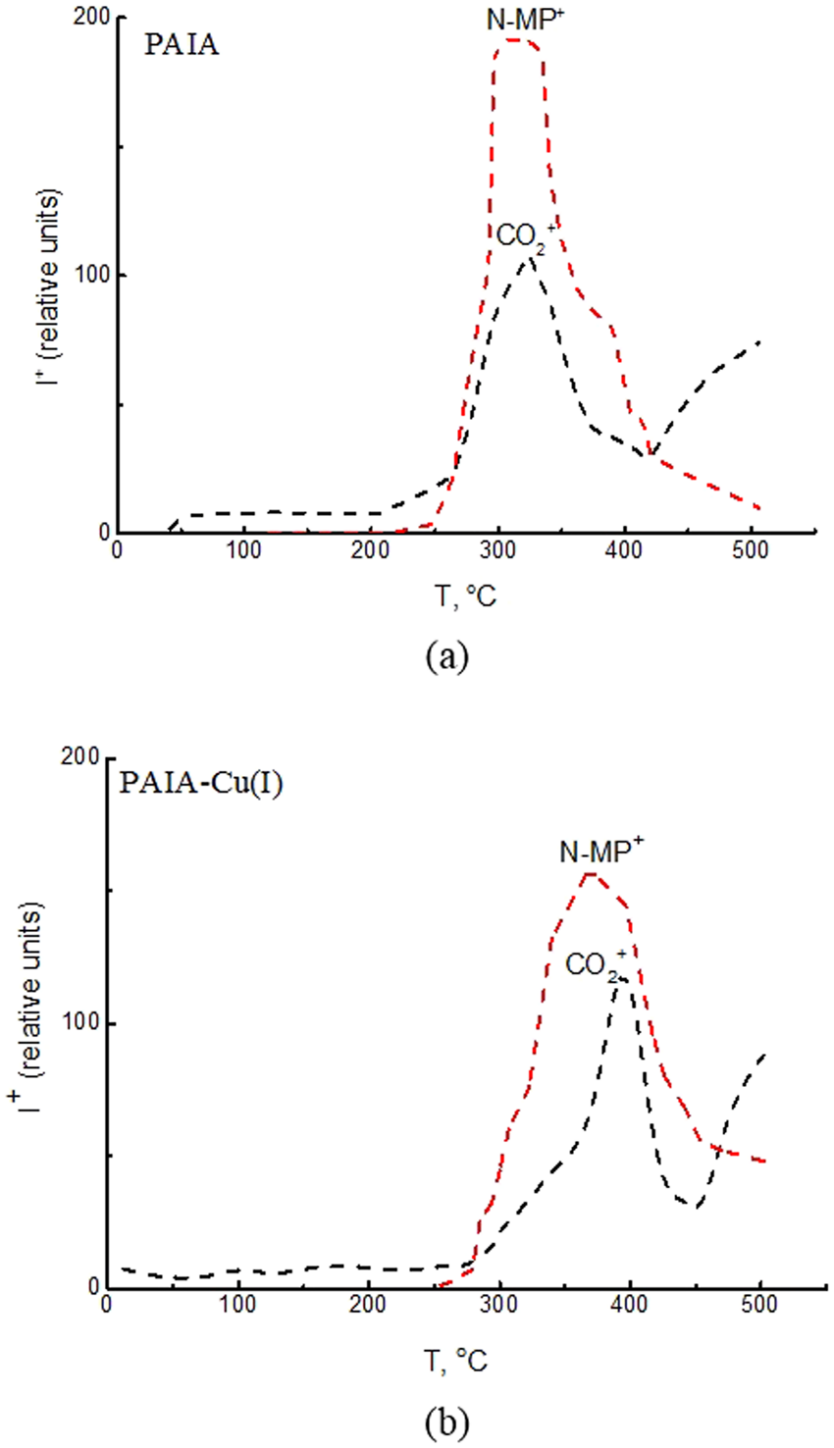
Temperature dependences of intensities of ion currents with *m/z* = 44 (black dash line, CO_2_^+^ ion) and *m/z* = 99 (red dash line, NMP^+^ ion) for PAIA (**a**) and PAIA–Cu(I) (**b**).

Mass spectrometric analysis of composition of the vapors appearing above the PAIA and PAIA-Cu(I) samples was carried out to confirm the presence of *N*-methylpyrrolidone (NMP) and its subsequent evaporation in the process of heating the polymer films. Heating rate was approximately 2 K/min. Figure 1 shows temperature dependences of intensities of CO_2_^+^ and NMP^+^ ion currents for the PAIA and PAIA-Cu(I) films. No other ions (except atmospheric background (N_2_^+^, O_2_^+^, Ar^+^)) were found in the mass spectra. Similar patterns of CO_2_^+^ and NMP^+^ ion currents were observed for both films. The peak with *m*/*z* = 99 corresponds to direct ionization of NMP. NMP begins to evaporate from the films in registerable amounts at ~250 °C. The peak with *m*/*z* = 44 corresponds to direct ionization of CO_2_ molecules that come from various sources (e.g., atmospheric background, thermal destruction of polymer film, and dissociative ionization of NMP).

At the beginning of the experiment (up to 250 °C), only CO_2_^+^ ions are detected in mass spectra; these particles appear as a result of ionization of atmospheric carbon dioxide. The CO_2_^+^ ion current observed in the range from 250 °C to 450 °C origins from atmospheric background and from dissociative ionization of NMP. When the NMP^+^ ion current starts to decrease, the CO_2_^+^ ion current increases, because now it comes from all three sources. Thermal decomposition of the film begins at 450 °C. Therefore, both films have similar thermal stability.

**Figure 2:**
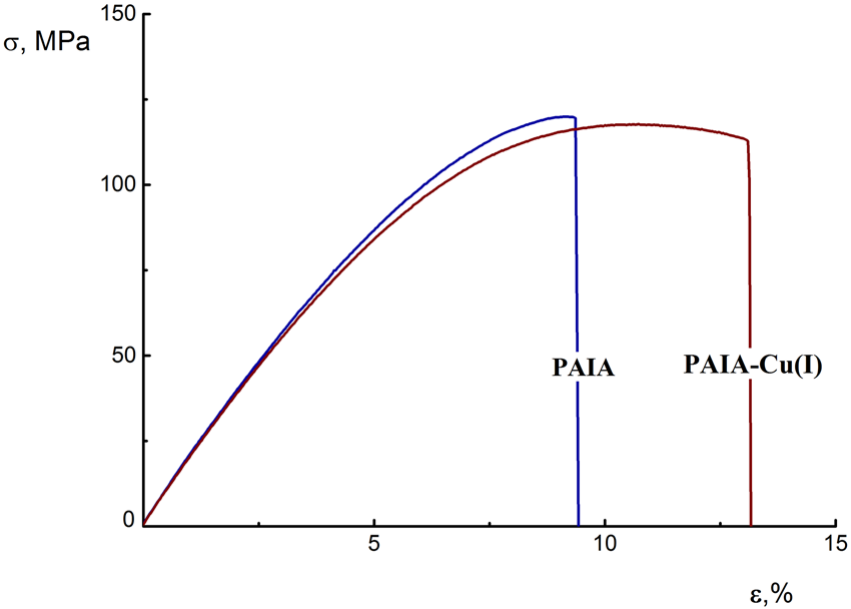
Deformation curves (dependence of strength (*σ*) on strain (*ε*)) for PAIA (blue line) and PAIA-Cu(I) (dark red line) films.

Both laboratory researches and industrial applications require only membranes with good physico-mechanical properties. Mechanical characteristics, such as Young’s modulus *E*, yield stress *σ*_*y*_, tensile strength *σ*_*b*_, and ultimate strain *ε*_*b*_, were measured for the PAIA and PAIA-Cu(I) membranes at room temperature under the uniaxial extension conditions. All the membrane samples demonstrated similar deformation behavior, namely, the tendency to realize plastic extension with the necking process. The well-defined yield point can be seen on stress-strain curves (Fig. 2). In both cases, film rupture occurs immediately after reaching the yield point of the material.

Table 1 lists the data on mechanical properties of the PAIA and PAIA-Cu(I) membranes. Formation of the polymer-metal complex with Cu(I) does not affect the E, σ_y_, and σ_b_ values of the film. Thermally stimulated physical transition was registered during thermo-mechanical experiments involving both films at 185 °C, which coincides with their glass transition temperature. This value agrees well with the corresponding value for the similar PAIs that have been studied previously^29^.

**Table 1:** Mechanical properties.

Membrane	Young’s modulus, *E*, GPa	Yield stress, σ_y_, MPa	Tensile strength, σ_b_, MPa	Ultimate strain, ε_b_, %	T_g_, °C	Density, g/cm^3^
PAIA	2.00 ± 0.07	118 ± 4	117 ± 5	9.5 ± 0.5	185 ± 2	1.357 ± 0.006
PAIA-Cu(I)	2.03 ± 0.06	119 ± 4	116 ± 4	12 ± 1	185 ± 2	1.381 ± 0.008

Table 1 presents the data on membrane density measured by flotation method. The density of the PAIA-Cu(I) membrane increases as compared with the corresponding parameter of the PAIA membrane. This fact shows that formation of the polymer-Cu(I) complex leads to appearance of more compact structure than that of the native membrane.

**Figure 3:**
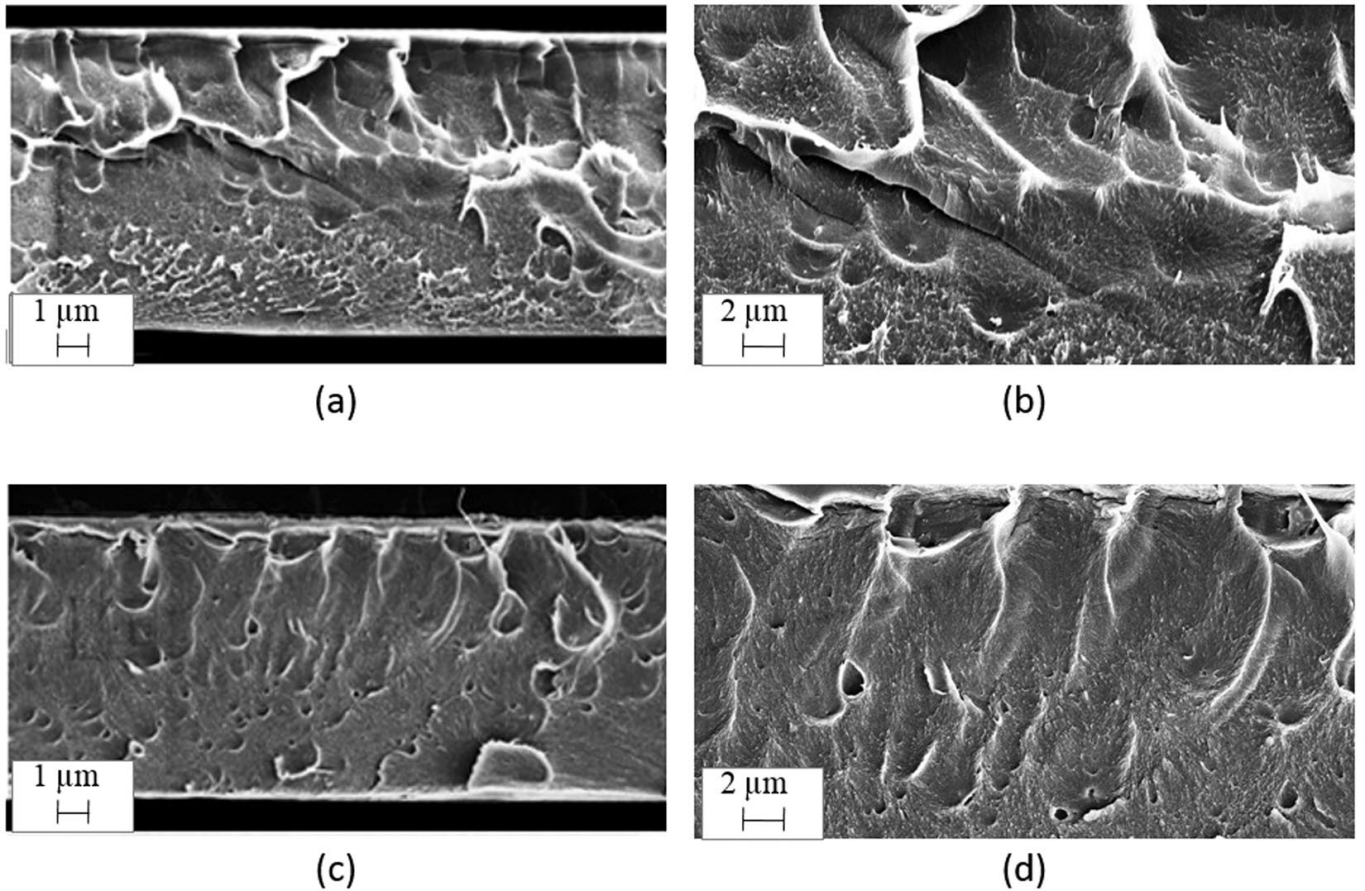
SEM images of membrane cross-section of (**a**,**b**) PAIA and (**c**,**d**) PAIA-Cu.

Scanning electron microscopy was used to study structure of the PAIA and PAIA-Cu(I) membranes. Figure 3 demonstrates images of the membrane cross-section before and after the formation of polymer-metal complex. Morphology of the PAIA cross-section is not uniform; tension bars and explicit traces of plastic deformations can be observed. Figure 3 shows that the PAIA-Cu(I) cross-section is more uniform and more dense as compared with the PAIA membrane. Cross-sectional images of the complete thickness of the membranes justify this fact (Fig. 3a,c). It was established that thickness of membranes prepared in the same conditions differed as follows 27 µm for PAIA and 23 µm for PAIA-Cu(I).

Table 2 shows the EDS data on elemental composition of membrane samples. It was established that *C*, *N*, and *O* contents are comparable to the corresponding calculated values. The presence of insignificant sulfur impurities is evidently related to traces of sulfolane, which was used as a solvent in the synthesis of the [2,2′-biquinoline]-6,6′-diyldimethanamine monomer. Experimentally measured copper content in the polymer‒Cu(I) complex corresponds to the calculated one.

**Table 2:** EDS еlemental analysis of membrane cross-sections.

Sample	C, wt%	N, wt%	O, wt%	S, wt%	Cl,wt%	Cu, wt%	Total,wt%
PAIA	70.52	8.82	20.46	0.20	0.00	0.00	100.0
PAIA-Cu(I)	71.16	7.20	20.41	0.21	0.31	0.71	100.0

The wettability of the membranes is an important characteristic for estimation of the affinity of the membrane materials to liquids. Contact angles of water (surface tension of 72.4 mN/m) and ethanol (surface tension of 21.4 mN/m) were determined by sessile drop method and presented in Table 3.

**Table 3:** Contact angles of the membranes.

Sample	Water, °	Ethanol, °
PAIA	52.7	19.2
PAIA-Cu(I)	60.2	22.0

Contact angles of water and ethanol (hydrophilic liquids) equal to 52.7 and 19.2 respectively for PAIA, which indicates on hydrophilic nature of membrane surface. It was found that formation of polymer-metal complex PAIA-Cu(I) leads to increase of contact angles for liquids under study. It means that the surface of PAIA-Cu(I) become more hydrophobic and worse wetted of hydrophilic liquids as compared PAIA.

### Transport properties

Mass transfer of two organic liquids (methanol and hexane) through the PAIA and PAIA-Cu(I) membranes was studied using sorption, desorption, and pervaporation tests. Transport properties of the membranes strongly depend on physicochemical properties of components of a mixture and on character of interaction between membrane polymers and components of separated mixture. Table 4 presents some physical properties of the liquids used in our study. Methanol and hexane have almost similar boiling points, while their densities, molar volumes, viscosities, and solubility parameters differ significantly.

**Table 4:** Physical properties of methanol and hexane at 35 °С.

Liquid	MW	Density, g/cm^3^	Molar volume, cm^3^/mol	*Т*_*b*_, °С	Viscosity, mPa∙s	Solubility parameter, (J/cm^3^)^1/2^
Methanol	32.0	0.792	40.4	64.7	0.55	29.7
Hexane	86.2	0.655	132	68.0	0.30	14.9

### Sorption-desorption study

Sorption experiments involved immersion of the PAIA and PAIA-Cu(I) membrane samples into the individual liquid (methanol or hexane). Table 4 gives the results of sorption studies, i.e., the following physicochemical properties of polymer-liquid systems: equilibrium sorption degrees (*S*) and Flory–Huggins interaction parameters (*χ*′) for methanol and hexane, as well as diffusion coefficients (*D*) of methanol. Both membranes exhibit higher equilibrium sorption degrees for methanol than those for hexane. The *S* value of methanol is higher in the case of PAIA than in the case of its polymer-metal complex. The *S* value of hexane is small enough for PAIA, but becomes slightly higher for polymer-metal complex. It was found due to PAIA-Cu(I) has a little more hydrophobic surface as compared PAIA (Table 3) and higher affinity to wetting and solve of hydrophobic liquids like hexane and less affinity to hydrophilic liquids like methanol.

In the sorption and pervaporation processes, the polymer active centers of a membrane are able to interact with components of liquid mixture by means of van der Waals, dipole–dipole, and ion–dipole forces, or through hydrogen bonding^30^. Quantitatively, the polymer‒penetrant interaction can be expressed in terms of the Flory–Huggins parameter (*χ*′)^31^. Usually, stronger interaction between a polymer and a penetrant results in relatively small value of *χ*′, because the amount of penetrant inside the polymer is higher. On the contrary, lower affinity between a polymer and a penetrant results in high *χ*′ value.

As seen from Table 5, comparatively low value of the *χ*′ parameter is observed in the case of methanol, since methanol interacts with these membranes better than hexane. The *χ*′ parameter for methanol increases in going from PAIA to PAIA-Cu(I); this means that interaction between polymer and methanol becomes weaker. The opposite effect is observed for hexane.

**Table 5:** Physicochemical properties of the studied polymer-liquid systems at 20 °C.

Polymer	Sorption degree, g liquid/100 g polymer		Parameter *χ*′		Diffusion coefficient of methanol, cm^2^/s
	Methanol	Hexane	Methanol	Hexane	
PAIA	12.6	0.8	1.34	3.20	2.6·10^−11^
PAIA-Cu(I)	8.0	1.05	1.60	2.97	1.8·10^−11^

**Figure 4:**
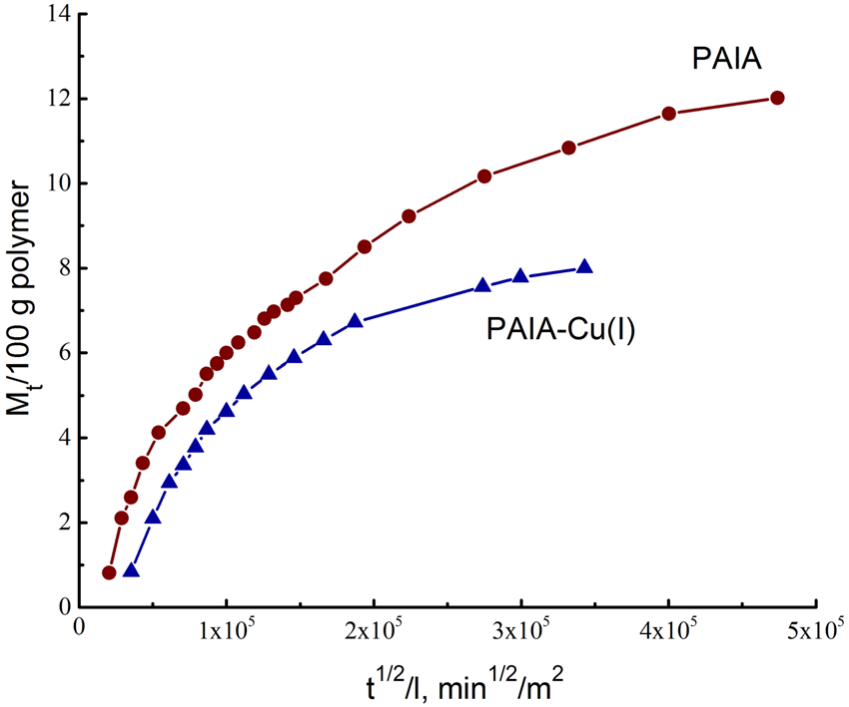
Kinetic curves of desorption of methanol from swollen PAIA (blue triangles) and PAIA-Cu(I) (dark red circles) membranes. The plots represent dependences of amount of methanol desorbed from 100 g polymer in time *t*.

Figure 4 shows kinetic curves of methanol desorption from swollen PAIA and PAIA-Cu(I) membranes. *M*_*t*_/100 is an amount of methanol desorbed from 100 g polymer in time *t*. The initial stage of desorption is governed by Fick’s law^32^. Here, migration of sorbate molecules is controlled by their diffusion inside membranes; linear parts of these curves can be used for estimation of effective diffusion coefficients of methanol. The values of effective diffusion coefficients were determined from the tangent values of the slopes of initial linear parts of desorption kinetic curves (Eq. 4). As seen from Table 5, the diffusion coefficient of methanol decreases after formation of the polymer-metal complex.

The physicochemical properties of polymer-liquid systems determined from sorption experiments have an influence on membrane transport properties in pervaporation. It should be noted that thermodynamic analysis of sorption in terms of pervaporation performance has been successfully carried out on the basis of the Flory-Huggins theory in several experimental and theoretical works^33–35^. This approach allows us to explain the pervaporation processes on the basis of the sorption–diffusion mechanism.

### Pervaporation of methanol–hexane mixture

Separation of methanol‒hexane mixtures is complicated by the presence of the azeotrope (about 27 wt% methanol) which is a limit for vapor–liquid based separation technologies, and also the wide immiscibility gap^34,36^. In the present work, the composition of the feed was chosen so that the process of pervaporation separation proceeded in a single-phase region at methanol concentration from 15 to 70 wt%, which includes the composition of the azeotropic mixture.

**Figure 5:**
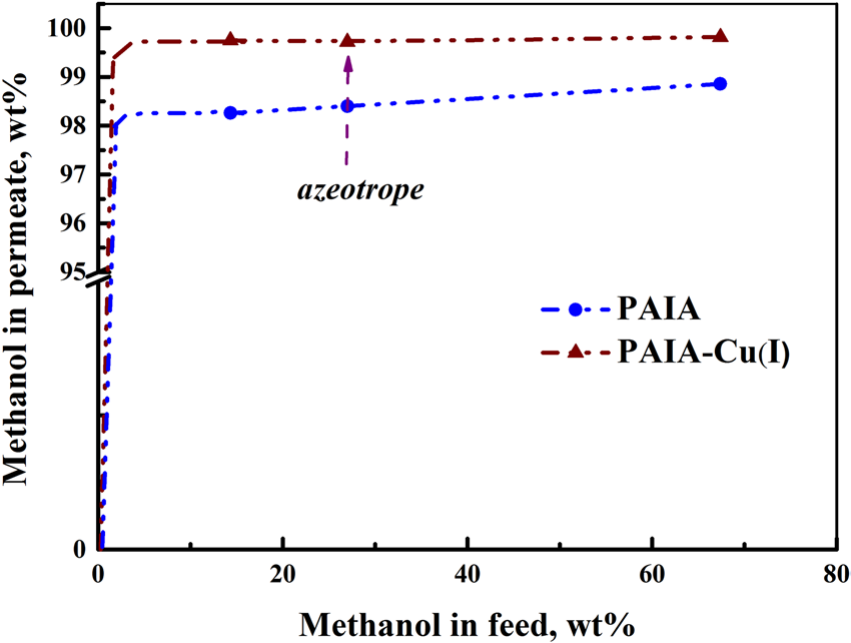
Dependence of methanol concentration in permeate on methanol concentration in feed for the PAIA (blue line) and PAIA-Cu(I) (dark red line) for pervaporation of methanol– hexane mixture at 40 °C.

Figure 5 shows the dependence of methanol concentration in permeate on methanol concentration in feed obtained in our pervaporation experiments. In pervaporation, the permeate is enriched with methanol and contains more than 99 wt% of methanol for PAIA-Cu(I).

**Figure 6:**
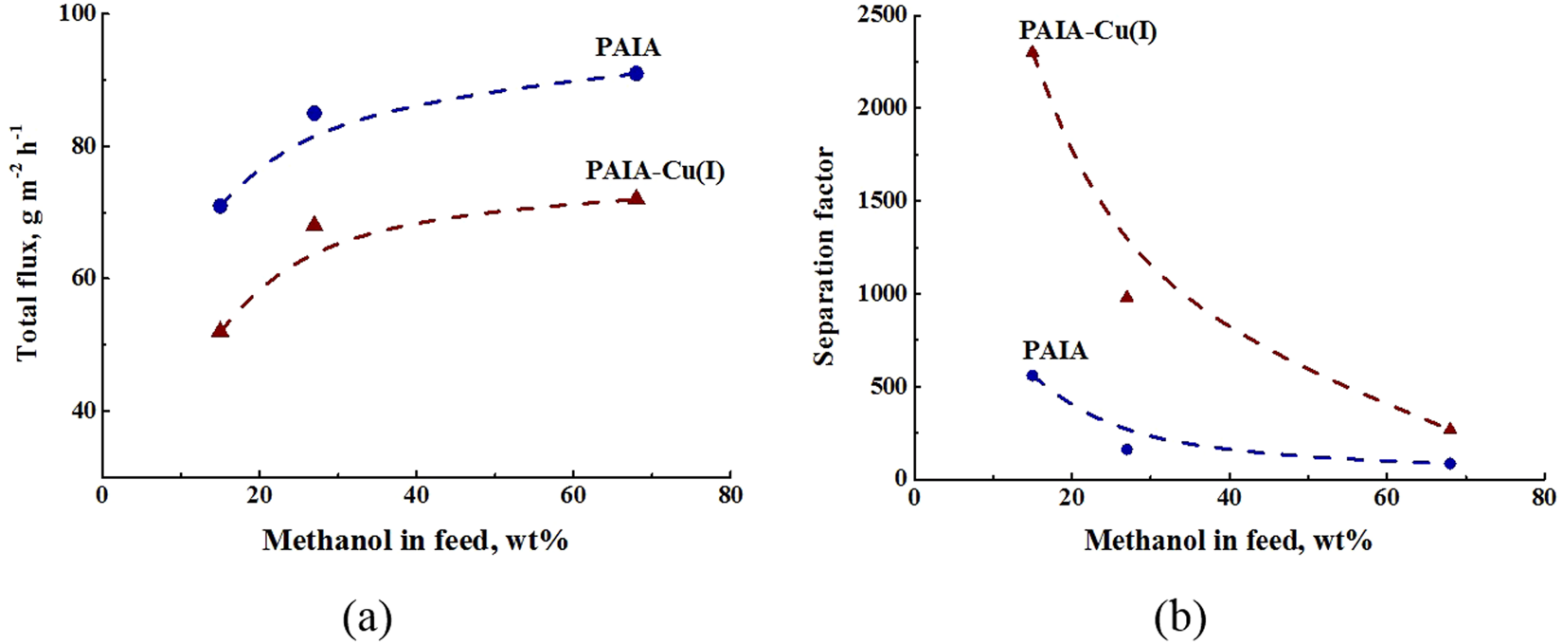
Dependences of (**a**) total flux and (**b**) separation factor (*α*_*Methanol/Hexane*_) on methanol concentration in feed for pervaporation of methanol‒hexane mixture through PAIA and PAIA-Cu(I) membranes, 40 °С.

Figure 6 shows dependences of total flux and separation factor (*α*_*Methanol/Hexane*_) on methanol concentration in the feed for the pervaporation of methanol‒hexane mixture through the PAIA and the PAIA-Cu(I) membranes at 40 °С. Both membranes are preferably permeable to methanol. As seen from Fig. 6b, the separation factor decreases with an increase of methanol concentration in feed. The PAIA-Cu(I) membrane exhibits higher separation factors for all feed compositions as compared to those of the initial PAIA. Because of the mechanism of pervaporation, the diffusion ability of the penetrant molecules plays a great role in mass transfer. Membrane base on polymer-metal complex PAIA-Cu(I) has more compact structure which effects on smaller diffusion coefficient and more selective but more longer permeation of the penetrants. Thus, total flux through PAIA is higher than that in the case of polymer-metal complex for all feed compositions and increases with increasing methanol concentration in feed.

**Figure 7:**
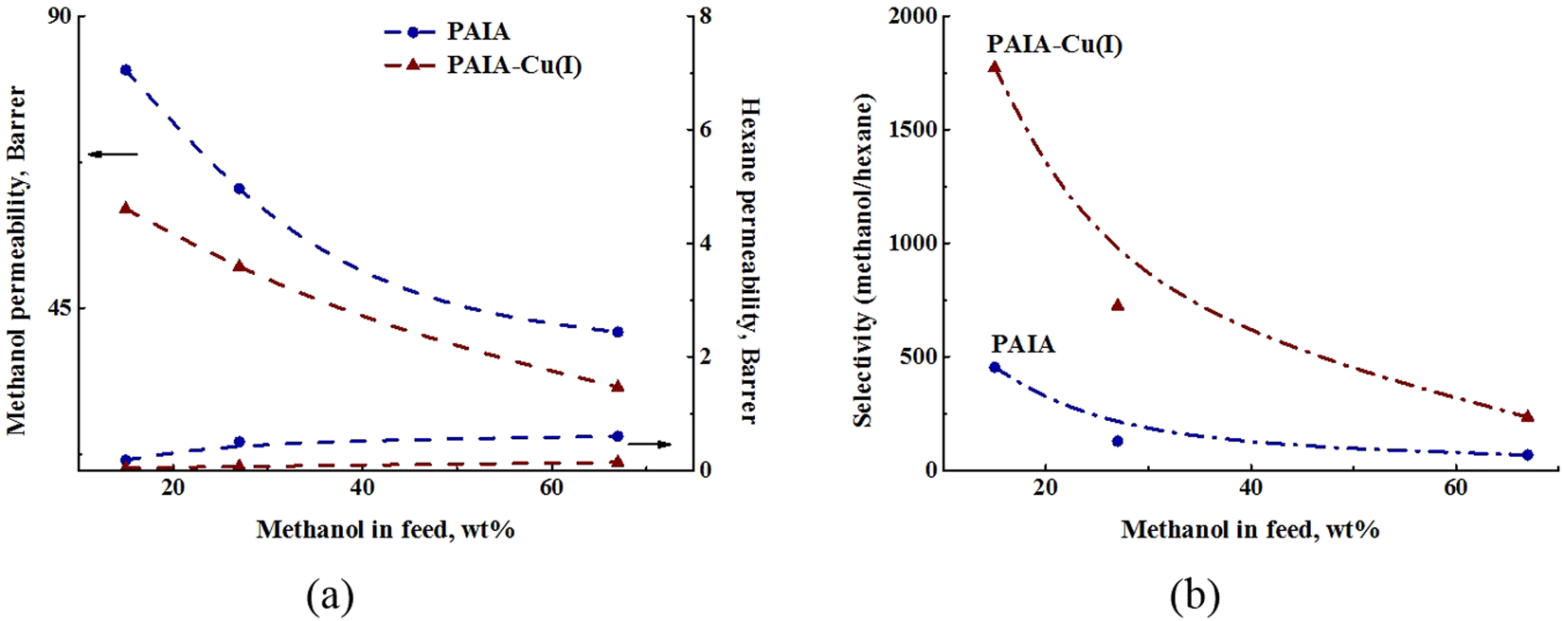
Dependences of (**a**) methanol and hexane permeability and (**b**) selectivity (*β*_*Methanol/Hexane*_) on methanol concentration in feed for the pervaporation of methanol‒hexane mixture through PAIA and PAIA-Cu(I) membranes, 40 °С.

To normalize the properties of membranes with respect to driving forces (partial vapor pressure), the approach of Baker *et al*.^36^ was used. The results of pervaporation experiments allow calculating the permeability (in addition to the flux) and selectivity (in addition to the separation factor). Figure 7a demonstrates dependences of permeabilities of methanol and hexane on methanol concentration in feed for the pervaporation of methanol‒hexane mixture through PAI-PAA and PAI-PAA-Cu(I) membranes. When the effects of driving force and operating conditions were eliminated, different behavior was observed. In contrast to the total flux of permeate (Fig. 5a), which consists mostly of methanol, methanol permeability decreases as methanol concentration in feed increases. At the same time, hexane permeability slightly increases, and this parameter is low for both studied membranes. Methanol permeability is high at low methanol concentration in feed due to high affinity of the films toward methanol. Hexane permeability, on the contrary, becomes higher at high methanol concentration in feed, when the film becomes significantly swollen, and thus penetration of large hexane molecules is facilitated.

Figure 7b demonstrates dependence of membrane selectivity (*β*_Methanol/Hexane_) on methanol concentration in feed. Selectivity is defined as the ratio of methanol permeability to hexane permeability. Figure 6b shows that reduction in selectivity results from decreasing methanol permeability with increasing methanol concentration in feed. In general, selectivity curves in Fig. 6b are similar to those of separation factor in Fig. 5b. However, selectivity values are lower than the values of separation factor.

Analysis of normalized values of permeability and selectivity shows that PAIA-Cu(I) membrane is less permeable but significantly more selective than PAIA membrane; this result is consistent with the data on the flux and separation factor.

Special attention was paid to the pervaporation separation of azeotropic mixture (methanol: hexane ≈27: 73 wt%)^15^. In order to make an objective evaluation of membrane transport properties, the pervaporation separation index (*PSI*) was calculated^37^. Table 5 lists the data on total flux, separation factor, and *PSI* of our membranes; these parameters were compared with the literature data on Nafion hollow fiber membrane based on branched fluorocarbons and containing side chains with end sulfo groups^21^. Total flux of PAIA-Cu(I) membrane decreases slightly as compared to that of the PAIA membrane. However, the separation factor of the PAIA-Cu(I) membrane increases by six orders of magnitude in comparison with the corresponding parameter for pure PAIA. These results clearly demonstrate that the PAIA-Cu(I) membrane is more promising of the two for separation of methanol‒hexane azeotropic mixture. The use of PAIA-Cu(I) complex makes it possible to separate the azeotropic mixture with a separation factor of 980 and *PSI* equal to 66.6; thus, this material is significantly more effective than Nafion. The total flux of the new membrane is noticeably lower than that of Nafion hollow fiber membrane. However, it should be taken into account that Table 6 lists the data for dense PAIA-Cu(I) membrane with a thickness of 20 μm. For industrial applications, it is necessary to develop a composite membrane with a thin (~2 μm) PAIA-Cu(I) layer that will be able to increase flux and PSI by one order of magnitude.

**Table 6:** Comparison of transport properties in pervaporation of azeotropic methanol‒hexane mixture (27:73 wt%), 40 °С.

Membrane	Total flux, kg/m^2^h	Separation factor	*PSI*
PAIA	0.085	162	13.7
PAIA-Cu(I)	0.068	980	66.6
Nafion^21^	0.58	17	9.8

## Conclusions

Two novel membranes were prepared on the basis of polyheteroarylene with 2,2′-biquinoline-6,6′ units and its complex with Cu(I). The polymer was synthesized with the use of a new bifunctional monomer [2,2′-biquinoline]-6,6′-diyldimethanamine obtained from the corresponding dicarbaldehyde by the Leuckart–Wallach reaction. SEM analysis of the PAIA and the PAIA-Cu(I) membranes and measurements of their density showed that the polymer-metal complex exhibits more compact structure than the initial polymer. Transport properties of these membranes toward methanol‒hexane mixture were studied by sorption, desorption, and pervaporation tests. The equilibrium sorption degrees of both membranes were higher in the case of methanol than in the experiments involving hexane. The sorption degree of methanol decreases when the polymer–metal Cu(I) complex is used. In pervaporation of methanol ‒ hexane mixture, both membranes demonstrate preferential permeability for methanol. Total flux through the PAIA membrane is higher than that through the polymer-metal complex for all feed compositions; this result is caused by better sorption activity of the membrane toward methanol and lower density of the initial membrane. It was found that the PAIA-Cu(I) membrane exhibits higher separation factors for all feed compositions as compared with those of the initial PAIA. In pervaporation of the azeotropic methanol ‒ hexane mixture, the separation factor of the PAIA-Cu(I) membrane increases by six orders of magnitude in comparison with that of pure PAIA sample.

The intrinsic properties of the penetrant−membrane system were evaluated using permeability and selectivity values of both membranes. The dependence of methanol permeability on methanol concentration in feed shows the opposite behavior to the corresponding dependence of the flux. The analysis of normalized values of permeability and selectivity shows that the PAIA-Cu(I) membrane is less permeable, but has significantly higher selectivity than the PAIA membrane; this is consistent with the data on the flux and separation factor. These results clearly demonstrate that the PAIA-Cu(I) membrane is more promising than the pure PAIA membrane for separation of azeotropic methanol‒hexane mixture with a separation factor of 980 and *PSI* equal to 66.6.

## Materials and Methods

### Materials

Sodium sulfate, hydroxylamine hydrochloride, copper(I) chloride, chloralhydrate, sulfuric acid, sulfolane, *N*-methylpyrrolidone (NMP), powdered copper, selenium dioxide, formic acid, propylene oxide, *p*-toluidine, ammonium carbonate, nickel formiate, and hydrochloric acid were obtained from commercial suppliers and used without further purification. Thionyl chloride was distilled, and the fraction boiling at 75.5 °C was taken off. N,N-dimethylformamide was dried over calcium hydride and distilled in vacuum at 0.1 mm Hg, bp. 56 °C. Methylene-bis-anthranilic acid was synthesized as described in^38,39^.

Bistrimellitimide dicarboxylic acid was synthesized as described in^26^. Methanol and hexane of chemically pure (CP) grade were purchased from Vekton (Russia) and used as received.

### Synthesis of PAIA-Cu(I)

#### Synthesis of [2,2′-biquinoline]-6,6′-diyldimethanamine

**Figure 8:**
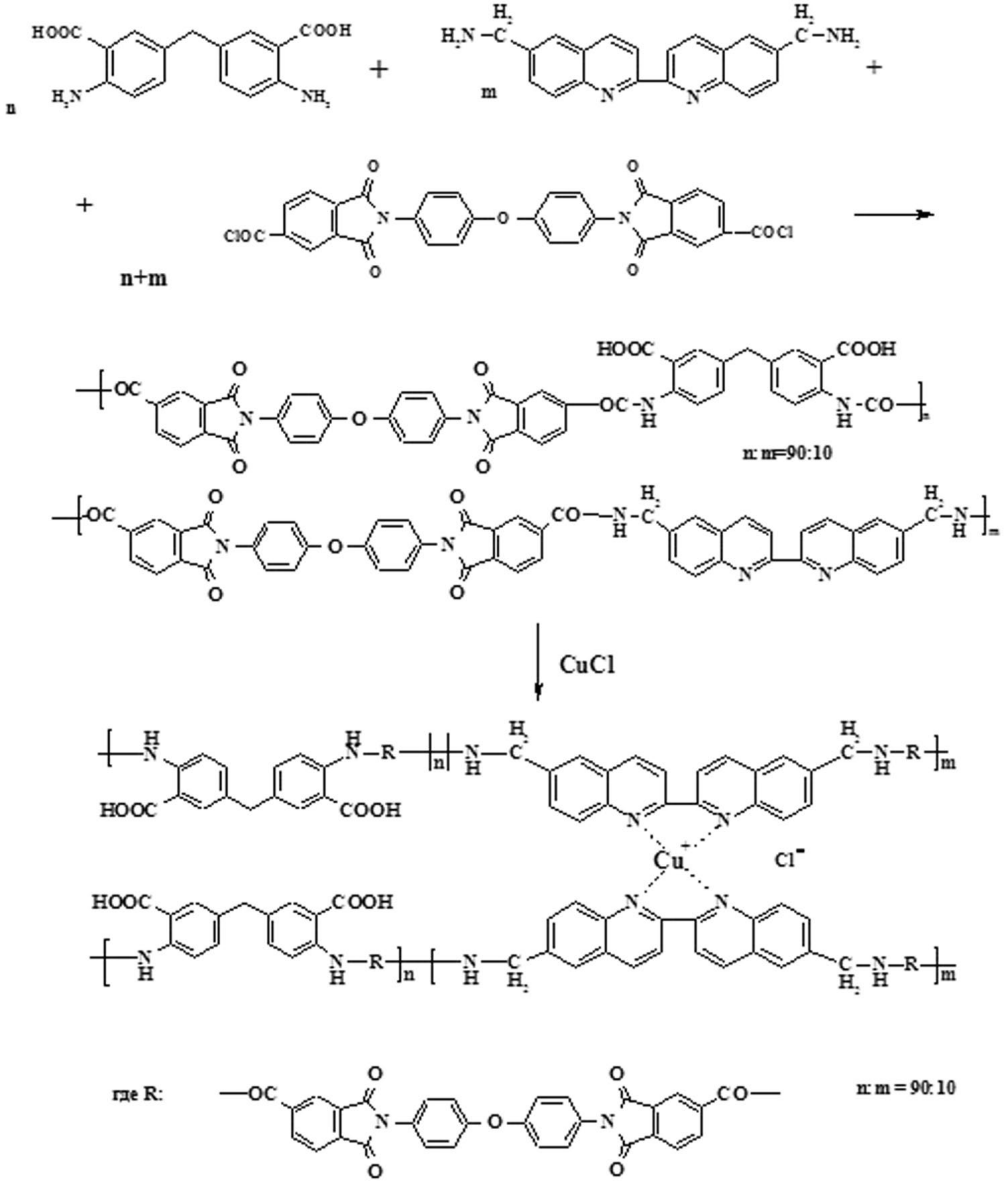
Scheme of PAIA-Cu(I) synthesis.

[2,2′-biquinoline]-6,6′-diyldimethanamine was synthesized by the Leuckart-Wallach reaction^39^ from [2,2′-biquinoline]-6,6′-dicarbaldehyde. ^1^*H* NMR (CF_3_COOH-D_2_O, δ,ppm): 3.35 (s, 4 H), 7.0 (d, 2H), 7.15 (s, 2H), 7.35 (d, 2H), 7.45 (d, 2H), 7.85 (d, 2H) (Fig. 8).

#### Synthesis of PAIA

A two-neck round-bottom flask equipped with a stirrer was charged with 0.257 g (0.0009 mol) of methylene-bis-anthranilic acid, 0.0314 g (0.0001 mol) of [2,2′-biquinoline]-6,6′-diyldimethanamine, and 7.5 mL of NMP; the mixture was stirred until diamines dissolved completely. Then the solution was cooled down to −15 °C. Bis-trimellitimidedicarboxylic acid dichloroanhydride (0.603 g, 0.00103 mol) was added to the cooled solution. After the suspension was stirred at −15 °C for 50 min, the flask was allowed to warm up to room temperature, and propylene oxide (0.05 mL) was added. Then the mixture was stirred at room temperature for 5 h.

^1^*Н* NMR (*DMF-d7*δ, ppm): 7.22 (d, 2 H), 7.57 (d, 2 H), 8.54 (s, 2 H) – methylene-bis-anthranilic fragment, 7.33 (d, 4 H), 7.67 (d, 4 H) -diphenyloxide fragment, 8.79 (d, 2 H), 8.45 (d, 2 H) – phthalimide fragment, 7.35 (d, 2 H), 7.45 (d, 2 H)- nitrogen containing cycle of biquinoline fragment, 7.0 (d, 2 H), 7.8 (d, 2 H)– benzene ring of biquinoline fragment, 3.35 (s, 4 H) methylene group.

#### Preparation of PAIA-Cu(I)

The PAIA-Cu(I) complex was obtained by adding the equimolar amount of copper (I) chloride dissolved in NMP to the solution of PAIA. The complex formation was carried out in a flask with a stirrer for a few hours at room temperature. It should be emphasized that formation of metal-polymer complex is accompanied by a marked increase in viscosity of polymer solution. The reason for this is intermolecular crosslinking of PAIA chains.

### Preparation of dense membranes

PAIA and PAIA-Cu(I) dense nonporous membranes were obtained by casting 10 wt% of polymer solution in N-methylpyrrolidone onto a glass plate. The solvent was evaporated at 140 °C in air. The membranes were dried at 90 °C in vacuum until the constant weight was reached.

However, the experience showed that such membranes contain residual amounts of NMP solvent. In order to eliminate the influence of residual solvent (NMP) on membrane properties, the obtained membranes were subjected to the special treatment with methanol that displaces NMP and washes it out^23^. Membranes were immersed into methanol, left to stand for three days, then thoroughly washed with methanol, and dried at 40 ^о^С in vacuum for two weeks^40–42^. Comparison of the membrane weight before and after treatment with methanol and subsequent drying showed that the membranes contained about 8 wt% of residual solvent (NMP) with respect to membrane weight.

### Characterization

#### Scanning electron microscopy (SEM)

Membrane morphology was studied using a Zeiss SUPRA 55VP scanning electron microscope (SEM) (Carl Zeiss AG, Germany). Before the experiment, sample surface was coated with a 20 nm thick platinum layer; the procedure was carried out by cathode sputtering with the aid of a Quorum 150 setup (UK).

#### Energy dispersive microanalysis (EDS)

Elemental compositions of samples and their individual components were determined using an INCA Energy microanalysis system equipped with an X-Max 80 OXFORD detector (which is a part of a SUPRA 55VP microscope). Spectra were taken from the surface of film samples as well as from single points of the surface in order to identify phases in a sample.

#### Mass spectrometry

Mass spectrometric investigation was carried out using the Knudsen effusion technique combined with mass spectrometric analysis of the vapor composition with the aid of an MS 1301 mass spectrometer (Construction Department of Russian Academy of Science, Saint Petersburg, Russia). Ionization of the vapor species was performed by electron ionization, with the energy of the ionizing electrons being 25 eV. The samples were evaporated from an open gold effusion cell placed in a molybdenum block and heated using a resistance furnace. The temperature was measured with a Pt−PtRh thermocouple and stabilized with an accuracy of ±1 °C.

#### Mechanical properties

Mechanical characteristics of the films were determined at room temperature under uniaxial extension conditions using band-like samples 2 mm wide and 20 mm long. The film strips for the tests were cut with a surgeon knife in the special laboratory-made unit, which ensures formation of strips with a constant width and mutually parallel lateral edges.

The experiments were carried out using an AG-100kNX Plus universal mechanical test system (Shimadzu, Japan). The extension speed was 5 mm/min. In the experiments, the Young’s modulus *E*, yield stress *σ*_*y*_, tensile strength *σ*_*b*_, and ultimate strain *ε*_*b*_ values were determined. The temperatures of physical transitions in the films under study were determined by thermomechanical method with the help of a TMA 402 F1 Hyperion thermal analyzer (NETZSCH Gerätebau GmbH, Germany). The heating rate during the tests was 5 deg/min.

#### Membrane density determination

Membrane density (ρ) was estimated by the flotation method with the aid of a laboratory-made measurement unit; a mixture of toluene and carbon tetrachloride was used to equilibrate film samples at 20 °C (*ρ*_*toluene*_ = 0.867 g/cm^3^, *ρ*_*СCl4*_ = 1.594 g/cm^3^)^43^.

#### Contact angles

Contact angles were measured to estimate the nature of the membranes surface by sessile drop method using a Drop Shape Analyzer DSA 10 (KRÜSS, Germany) at 20 °C and atmospheric pressure.

#### Sorption study

Sorption experiments involved immersing membrane samples into an individual liquid (methanol or hexane) at atmospheric pressure, at 20 °С. The weight change was determined gravimetrically (measurement error ±10^−4^ g). The experiment was continued until the sample weight became constant, and equilibrium was reached.

The equilibrium sorption degree (*S*, g liquid/100 g polymer) was calculated by the following equation:

1 $$S=\frac{{M}_{s}-{M}_{d}}{{M}_{d}}\cdot 100$$

where *M*_*s*_ is the weight of a swollen membrane in the equilibrium state, and *M*_*d*_ is the weight of a dry membrane.

After completion of sorption experiments, solvent desorption was carried out by exposing the samples to air at 20°С in the controlled environment (in the exsiccator containing molecular sieve absorber). Changes in sample weight as a function of time were recorded until equilibrium was reached. Kinetic curves of desorption *M*_*t*_*/M*_*∞*_ = *f* (*t*^*1/*2^*/l*) were plotted, where *M*_*t*_ is the amount of desorbed substance per time *t*, *M*_*∞*_ is the equilibrium amount of desorbed substance (calculated as a difference between the weight of a swollen membrane and the weight of a membrane dried to constant weight), and *l* is the membrane thickness^44^. The effective diffusion coefficient *D* was calculated by the following equation:

2 $$D=\frac{\pi }{16}{(\tan \beta )}^{2}$$

where *tanβ* is the tangent of the slope of initial linear part of desorption kinetic curve (when *М*_*t*_*/М*_*∞*_ < 0.4).

The data on membrane sorption and density were used for calculation of *φ*_*2*_ (the volume fraction of a polymer in the swollen membrane):

3 $$\phi =\frac{1}{1+\frac{{\rho }_{2}}{{\rho }_{1}}\cdot {S}_{w}}$$

where *ρ*_1_ and *ρ*_2_ are the solvent and polymer densities, respectively.

To characterize solubility of polymer in a given liquid, the equation from the Flory-Huggins theory was used^31,45^:

4 $$\mathrm{ln}\,{a}_{1}=\,\mathrm{ln}(1-{\phi }_{2})+{\phi }_{2}+\chi ^{\prime} \cdot {\phi }_{2}^{2}$$

The interaction parameter (χ′) was calculated from the swelling data assuming that the system can be considered as a dilute solution (where activity of the solvent is close to one (*ln α*_1_ = 0)), by the following formula^46^:

5 $$\chi ^{\prime} =\frac{-[\mathrm{ln}(1-{\phi }_{2})+{\phi }_{2}]}{{\phi }_{2}^{2}}$$

#### Pervaporation test

The transport properties in pervaporation of methanol–hexane mixture were measured using a laboratory cell having an effective membrane area of 14.8 cm^2^ at 40°С under stirring. The downstream pressure values not exceeding 10^−2^ mm Hg were maintained. The permeate was collected in a trap cooled with liquid nitrogen, weighted, and analyzed. Permeate composition was determined using a ≪Chromatec–Crystal 5000.2≫ chromatograph (Chromatec, Russia) equipped with a thermal conductivity detector.

The results of pervaporation experiments were used to calculate the total permeation flux and separation factor^47,48^. The separation factor *α*_*ij*_ was defined using the following equations:

6 $${\alpha }_{ij}=({Y}_{i}/{Y}_{j})/({X}_{i}/{X}_{j})$$

where subscripts *i* and *j* refer to methanol and hexane, respectively; *Y* and *X* are the weight fractions of the corresponding components in the permeate and feed, respectively.

The total flux through membrane (*J*) was determined as an amount of liquid penetrated through membrane area per unit time. To compare membranes with different thicknesses (*l*), which varied from 18 to 24 µm, the value of normalized flux (*J*_*n*_) was used. *J*_*n*_ is the flux through membrane with 20 µm thick calculated as follows:

7 $${J}_{n}=J\cdot l/20$$

Membrane efficiency was estimated using the pervaporation separation index (*PSI*)^49^ by the following equation:

8 $$PSI=J\cdot (\alpha -1)$$

With the purpose of estimating intrinsic properties of a penetrant-membrane system, permeability and selectivity were calculated^36^. Membrane permeability (*P*_*i*_) can be determined as a flux of a component normalized for membrane thickness and driving force; it was calculated using the following equation:

9 $${P}_{i}={j}_{i}\frac{l}{{p}_{i0}-{p}_{il}}$$

where *j*_*i*_ is the molar flux of component *i* (cm^3^ (STP)/cm^2^ s), and *p*_*i*0_ and *p*_*il*_ are the partial pressures of component *i* on both sides of the membrane (0 stands for the surface on the feed side, and *l* stands for the surface on the feed side). Permeability was expressed in Barrer units (1 Barrer = 1 · 10^−10^ (cm^3^ (STP)·cm/cm^2^ s·cmHg).

Membrane selectivity *β*_*ij*_ was defined as a ratio of the permeabilities:

10 $${\beta }_{ij}=\frac{{P}_{i}}{{P}_{j}}$$

## References

[1] Yampolskii, Y., Pinnau, I. & Freeman, B. D. *Materials Science of Membranes for Gas and Vapor Separation*, 10.1002/047002903X (2006).

[2] Baker, R. W. *Membrane Technology and Applications*. (Wiley 2012).

[3] Feng X, Huang RYM (1997). Liquid Separation by Membrane Pervaporation: A Review. Ind. Eng. Chem. Res..

[4] Jyoti G, Keshav A, Anandkumar J (2015). Review on Pervaporation: Theory, Membrane Performance, and Application to Intensification of Esterification Reaction. J. Eng..

[5] Crespo, J. G. & Brazinha, C. Pervaporation in *Pervaporation*, *Vapour Permeation and Membrane Distillation: Principles and Applications* (Woodhead Publishing, 2015).

[6] Ulbricht, M. Advanced functional polymer membranes. *Polymer* (*Guildf*). (2006).

[7] Smitha B, Suhanya D, Sridhar S, Ramakrishna M (2004). Separation of organic-organic mixtures by pervaporation: a review. J. Memb. Sci..

[8] Ong YK (2016). Recent membrane development for pervaporation processes. Prog. Polym. Sci..

[9] Baker RW (2010). Research needs in the membrane separation industry: Looking back, looking forward. J. Memb. Sci..

[10] Brüschke, H. E. A. & Wynn, N. P. In *Encyclopedia of Separation Science* 1777–1786 (Elsevier, 2000).

[11] Chopade, S. P. & Mahajani, S. M. In *Encyclopedia of Separation Science* 3636–3641 (Elsevier, 2000).

[12] Babalou, A. A., Rafia, N. & Ghasemzadeh, K. *Pervaporation*, *Vapour Permeation and Membrane Distillation*. (Woodhead Publishing, 2015).

[13] Widagdo S, Seider WD (1996). Journal review. Azeotropic distillation. AIChE J..

[14] Cai F, Xiao G (2015). Liquid + liquid) extraction of methanol from alkanes using dialkylphosphate-based ionic liquids as solvents. J. Chem. Thermodyn..

[15] Hölscher IF, Schneider GM, Ott JB (1986). Liquid-liquid phase equilibria of binary mixtures of methanol with hexane, nonane and decane at pressures up to 150 MPA. Fluid Phase Equilib..

[16] Forman, S. E. Azeotropic distillation of methanol and acetone with hexane. (1952).

[17] Genduso G, Amelio A, Luis P, Van der Bruggen B, Vreysen S (2014). Separation of methanol-tetrahydrofuran mixtures by heteroazeotropic distillation and pervaporation. AIChE J..

[18] Ohya, H., Kudryavsev, V. V. & Semenova, S. I. *Polyimide Membranes: Applications*, *Fabrications and Properties*. (CRC Press, 1997).

[19] Jiang LY, Wang Y, Chung T-S, Qiao XY, Lai J-Y (2009). Polyimides membranes for pervaporation and biofuels separation. Prog. Polym. Sci..

[20] Pulyalina AY, Polotskaya GA, Toikka AM (2016). Membrane materials based on polyheteroarylenes and their application for pervaporation. Russ. Chem. Rev..

[21] Cabasso I (1983). Organic liquid mixtures separation by permselective polymer membranes. 1. Selection and characteristics of dense isotropic membranes employed in the pervaporation process. Ind. Eng. Chem. Prod. Res. Dev..

[22] Zhou F, Koros WJ (2006). Study of thermal annealing on Matrimid® fiber performance in pervaporation of acetic acid and water mixtures. Polymer (Guildf)..

[23] Pulyalina A (2017). Novel approach to determination of sorption in pervaporation process: A case study of isopropanol dehydration by polyamidoimideurea membranes. Sci. Rep..

[24] Pulyalina AY (2015). Ethanol purification from methanol via pervaporation using polybenzoxazinoneimide membrane. Fuel Process. Technol..

[25] Polotskaya G (2005). Gas transport properties of polybenzoxazinoneimides and their prepolymers. Polymer (Guildf)..

[26] Polotskaya GA (2003). Pervaporation membranes based on imide-containing poly(amic acid) and poly(phenylene oxide). J. Appl. Polym. Sci..

[27] Polotskaya G (2015). Structure and Gas Transport Properties of Polybenzoxazinoneimides with Biquinoline Units in the Backbone. Macromol. Symp..

[28] Pulyalina A (2017). Preparation and characterization of methanol selective membranes based on polyheteroarylene – Cu(I) complexes for purification of methyl tertiary butyl ether. Polym. Int..

[29] Goikhman MY (2016). Iridium metal–polymer complexes based on bipyridyl ligands. Polym. Sci. Ser. B.

[30] Prausnitz, J. M., Lichtenthaler, R. N. & Azevedo, E. G. *Molecular thermodynamics of fluid-phase equilibria*. (Prentice Hall PTR, 1998).

[31] Flory, P. J. *Principles of Polymer Chemistry*. (Cornell University Press, 1953).

[32] *Mass Transfer in Chemical Engineering Processes*. (InTechOpen, 2011).

[33] Naidu BVK, Aminabhavi TM (2005). Pervaporation Separation of Water/2-Propanol Mixtures by Use of the Blend Membranes of Sodium Alginate and (Hydroxyethyl)cellulose: Roles of Permeate−Membrane Interactions, Zeolite Filling, and Membrane Swelling. Ind. Eng. Chem. Res..

[34] Chovau S, Van der Bruggen B, Luis P (2012). Application of the mass-based UNIQUAC model to membrane systems: A critical revision. J. Chem. Thermodyn..

[35] Adoor SG, Prathab B, Manjeshwar LS, Aminabhavi TM (2007). Mixed matrix membranes of sodium alginate and poly(vinyl alcohol) for pervaporation dehydration of isopropanol at different temperatures. Polymer (Guildf)..

[36] Baker RW, Wijmans JG, Huang Y (2010). Permeability, permeance and selectivity: A preferred way of reporting pervaporation performance data. J. Memb. Sci..

[37] Huang, R. Y. *Pervaporation membrane separation processes*. (Elsevier, 1991).

[38] Kubota, M. & Hayashi, M. Preparation of diaminodiphenylmethanedicarboxylic acids. (1999).

[39] Pollard CB, Young DC (1951). The mechanism of the leuckart reaction. J. Org. Chem..

[40] Joly C, Le Cerf D, Chappey C, Langevin D, Muller G (1999). Residual solvent effect on the permeation properties of fluorinated polyimide films. Sep. Purif. Technol..

[41] Alentiev A, Yampolskii Y, Kostina J, Bondarenko G (2006). New possibilities for increasing the selectivity of polymer gas separating membranes. Desalination..

[42] Pulyalina AY, Toikka AM, Polotskaya GA (2014). Investigation of pervaporation membranes based on polycarbamide: Effect of residual solvent. Pet. Chem..

[43] Pientka Z (2013). Synthesis and characterization of polybenzoxazinone and its prepolymer using gas separation. Macromol. Chem. Phys..

[44] Wolińska-Grabczyk A (2007). Transport of liquid hydrocarbons in the polyurethane-based membranes. J. Memb. Sci..

[45] Teraoka, I. *Polymer Solutions*. *John Wiley & Sons* (2002).

[46] Kocherbitov V (2004). A new formula for accurate calculation of water activity in sorption calorimetric experiments. Thermochim. Acta..

[47] Koter S, Kujawska A, Kujawski W (2015). Modeling of transport and separation in a thermopervaporation process. J. Memb. Sci..

[48] Mulder, M. *Basic Principles of Membrane Technology*. (Springer Netherlands, 1996).

[49] Salehian, P. & Chung, T.-S. Thermally treated ammonia functionalized graphene oxide/polyimide membranes for pervaporation dehydration of isopropanol. *J. Memb. Sci.* **528**, 231–242 (2017).

